# Small airways disease and severe asthma

**DOI:** 10.1186/s40413-017-0153-4

**Published:** 2017-06-21

**Authors:** Tara F. Carr, Roula Altisheh, Myron Zitt

**Affiliations:** 1 Division of Pulmonary, Allergy, Critical Care and Sleep Medicine, Department of Medicine, University of Arizona, Tucson, Arizona USA; 2 Division of Allergy/Immunology, Department of Medicine, State University of New York, Stonybrook, NY, USA

## Abstract

The small airways of the lungs are commonly affected in pediatric and adult asthma. Small airways disease has been related to asthma control, severity, and risk of exacerbation. Diagnosis of small airways disease can be best made through evaluation of surgical lung specimens. Noninvasive techniques including spirometry, plethysmography, nitrogen washout, impulse oscillometry, and cross-sectional imaging have been utilized to assess and infer the extent of small airways disease in asthma and can be used longitudinally to assess response to treatment. Patients with small airways disease seem to benefit from inhaled asthma medications that have improved capacity to reach the distal lung compartment. This is especially important for patients with severe asthma, who rely upon high doses of inhaled corticosteroid and bronchodilators for asthma control. This review will describe the techniques which may be utilized to assess small airways disease, discuss the prevalence and characteristics of small airways disease in severe asthma, and highlight how small airway disease may complicate severe asthma treatment.

## Key points

1. Small airways disease is common in pediatric and adult asthma, particularly in those with more severe disease or more frequent symptoms.
2. While there is no noninvasive gold standard technique for the assessment or diagnosis of small airways disease in asthma, spirometry, plethysmography, nitrogen washout, fraction of exhaled nitric oxide, impulse oscillometry, and cross-sectional imaging can be used to infer the extent of small airways disease.
3. Treatment of asthma, particularly for those with more severe disease, should take into account the extent of small airways disease, and utilizing devices which optimize drug delivery to the small airways should be considered.

## Background

Asthma is a common, heterogeneous disorder characterized as a chronic inflammatory disease of the airways with bronchial hyperresponsiveness to a variety of stimuli, and variable airflow limitation that is often reversible either spontaneously or as a result of therapy [1]. Historically, asthma was understood to be a disease primarily of the large airways. However, autopsy specimens from individuals with fatal asthma reveal mucus plugging and inflammatory involvement of both the small and large airways [2]. In examining surgical lung specimens from patients living with chronic asthma, Hamid et al. [3], recognized an inflammatory process characterized by increased T cells, activated eosinophils and major basic protein in the small airways, which was similar to the inflammation seen in the central airways. This observation and those of Synek et al. [4] helped to confirm that the chronic inflammation which characterizes asthma involves the entire lung, from the large proximal to the small distal airways.

The extent to which disease of the small airways contributes to morbidity of asthma, particularly of severe asthma, is of significant interest. Challenges to implementing small airways assessments in the routine clinical setting and as part of severe asthma management include technical aspects of assessment and monitoring of small airways disease, and the impact of small airways on asthma therapeutic delivery and outcomes. The purpose of this review is to describe the techniques that may be utilized to assess small airways disease, to discuss the prevalence and characteristics of small airways disease in severe asthma, and to highlight how small airway disease may impact severe asthma treatment.

## Methods

We performed a literature review utilizing PubMed and other online resources, using search terms including “small airway”, “asthma”, “severe asthma”, “plethysmography”, “FeNO,” etc. Papers were reviewed for relevance and scientific merit.

## Definition of small airways disease

The small airways of the lung are defined as the bronchial passages less than 2 mm in diameter and located beyond the 7^th^ or 8^th^ generation of the tracheobronchial tree. These airways account for >98% of the cross sectional area of the lung and terminate with the alveolar sacs. These small airways have no cartilage to support their structure and are therefore more easily collapsed upon compression [5]. Disease of the small airways in asthma is described as cellular infiltration of bronchiolar walls, alveoli, and perivascular spaces [6, 7]; goblet cell hyperplasia of the epithelium [8]; collections of mucus and inflammatory cells obstructing the airway lumen [2]; smooth muscle thickening; and submucosal remodeling [9]. Physiologic characteristics of small airways obstruction include premature airway closure and air trapping, regional heterogeneity and exaggerated volume dependence of airflow limitation [10].

## Assessment and monitoring of small airways disease

The assessment and monitoring of the involvement of the small airways in asthma is challenging because of the relative inaccessibility of this region of the lung. The “gold standard” of small airways disease diagnosis has been the morphometry of resected lung tissue, but the invasiveness of this approach precludes its use clinically.

While early studies suggested that the small airways in normal individuals represents a “quiet zone” accounting for less than 10% of total airways resistance [11], more recent studies have placed greater importance on the distal airways. Wedged bronchoscopy, although mainly a research tool, because of its invasiveness and technical difficulty, has been used to obtain direct measurements of airways resistance in distal lung segments. Using this procedure, Yanai et al. [12] reported that total small airways resistance was 24% of total airway resistance in healthy adults and increased to 34% in asymptomatic newly diagnosed patients with asthma and to 51% in patients with severe asthma. Wagner et al. [13] demonstrated the small airways resistance in asymptomatic asthma patients with normal spirometry and normal plethysmographic airway resistance to be more than 7-fold higher than normal subjects. Further, peripheral airways resistance in patients with asthma is markedly increased with methacholine and histamine challenge [13, 14].

In order to best determine the clinical relevance of these findings, a non-invasive, reliable and reproducible means of assessing the distal airways and their response to therapy is necessary. While to date, a universally accepted assessment approach is lacking, tests that focus on the aforementioned clinical characteristics may be useful surrogates to detect and quantify small airways disease. A summary of tests that can be used to assess small airway disease in asthma is presented in Table 1.

**Table 1:** Techniques used for the assessment of small airways disease in asthma

Histologic	Functional	Radiographic
Lung biopsy	Spirometry FEF_25-75_ Plethysmography RV, RV/TLC, PC_20_, FVC/SVC Nitrogen washout Fraction of exhaled nitric oxide Impulse Oscillometry	High resolution computed tomography Magnetic resonance imaging with [3]He

### Evaluating functional parameters

#### Spirometry

For the measurement of dynamic lung volumes, spirometry is the most widely employed, non-invasive, and easy to perform procedure used worldwide to assess the airflow limitation associated with asthma. The forced expiratory volume in 1 s (FEV_1_) is generally accepted as the gold standard for clinically evaluating airways obstruction and assessing response to therapy. However, this measurement does not provide a comprehensive evaluation of the entire bronchial tree and is most reflective of abnormalities in the large and medium sized airways. The forced vital capacity (FVC)- the forced expiratory maneuver from total lung capacity (TLC) to residual volume (RV)- results in progressive volume-dependent airway closure and air trapping resulting in the concavity of the expiratory phase of the flow volume loop in patients with airflow limitation. The less effort-dependent forced mid expiratory flow between 25% and 75% (FEF_25-75_) can be computed from the flow volume loop and correlates highly, though non linearly with FEV_1_/FVC such that it decreases more steeply than this ratio at levels of mild airflow limitation. Thus, it is generally believed to be more reflective of small airways obstruction than is FEV_1_ or FEV_1_/FVC [15, 16]. FEF_25-75_ has been shown to be an early marker of small airways impairment in subjects with allergic rhinitis [17], and FEF_25-75,_ but not FEV_1,_ has been associated with asthma exacerbations [18]. Several studies have shown a good correlation between FEF_25-75_ and the high resolution computerized tomography (HRCT) finding of air trapping. Nonetheless the validity of the FEF_25-75_ measurement has been questioned as levels are influenced by large airways obstruction and volume changes and serial measures have been highly variable [19]. Further, FEF_25-75_ was found not to correlate with small airway inflammation as determined by bronchoscopically obtained lung biopsies [20], while data from the Severe Asthma Research Program showed a poor correlation with other measures of air trapping [21].

#### Plethysmography

Body plethysmography is a non-invasive procedure that provides static lung volume measurements related to air trapping and hyperinflation such as residual volume (RV), total lung capacity (TLC) and the RV/TLC ratio. Among them, RV has shown a closer relationship with changes in peripheral resistance, indicating that it could correlate with small airway function [22]. Indeed, RV is increased in the presence of premature airway closure and air trapping. Because TLC is commonly elevated in obstructive disease, the RV/TLC ratio should be evaluated concurrently; its elevation is considered the first step of hyperinflation [23] and is markedly increased in severe compared to non-severe asthmatics [24]. As RV/TLC inversely correlates reasonably well with FVC, in the absence of volume measurements, a reduction in FVC could be considered as a marker of air trapping. This may be underestimated in individuals with elevated TLC. The provocative concentration of methacholine causing a 20% fall in FEV1 (PC_20_) has also been described by several investigators as being a useful marker of air trapping [25], while greater sensitivity has been reported when both FEF_25-75_ and FEV1 were used together to evaluate the response to methacholine. Finally, the difference between slow inspiratory vital capacity (SVC) and FVC and the FVC to SVC ratio may be surrogate markers of the collapsibility of small airways [26]. Plethysmography is a useful tool in both adult and pediatric populations [27, 28].

#### Other respiratory maneuvers

In addition to the more familiar spirometric techniques, several other methods can be used to infer the presence of small airways dysfunction. Gas dilution maneuvers are noninvasive techniques that can estimate lung volume through calculations of the change in a known inhaled concentration of gas, upon exhalation. In the nitrogen washout test, exhaled nitrogen is measured after a single breath of nitrogen is inhaled to TLC. The rate and amount of nitrogen washout from the lungs can distinguish between ventilation inhomogeneity originating in the distal airways (due to premature closure of airways) from the more central airways. Inhomogeneity measured by these techniques correlates with asthma characteristics of recurrent exacerbations [29] and poor control [30].

Nitric oxide (NO) is an endogenous regulatory molecule that is widely distributed throughout the body. Its synthesis is mediated by a family of enzymes, the nitric oxide synthases (NOS). Inducible NOS-derived NO is predominantly produced in airway epithelial cells throughout the lung and increase in asthma during Th2 driven inflammation, which is generally eosinophilic in nature. Fractional exhaled nitric oxide (FeNO) measurement is therefore considered to be a useful non-invasive biomarker reflecting Th2-driven airway inflammation throughout the lung. Recommendations from the American Thoracic Society/European Respiratory Society for the measurement of nitric oxide have been published [31]. However, to be a useful marker of small airways disease, the contribution of FeNO from the distal airways (alveolar nitric oxide) needs to be accurately distinguished from that of the proximal, larger airways [32, 33]. While mathematical equations have been developed to differentiate alveolar nitric oxide from FeNO, the accuracy and utility remains controversial.

Impulse oscillometry (IOS) is an effort independent measurement of small airway function, and is particularly useful in the pediatric setting, as it requires only passive cooperation from the patient [34] to measure pulmonary impedance, the total force needed to propagate a pressure wave through the pulmonary system and its components, pulmonary resistance, the energy required to propagate the pressure wave through the airways, and reactance, the amount of recoil generated against that pressure wave. Measurements of resistance at varying oscillation frequencies of the pressure wave can identify obstruction of small airways [35, 36], and the related characteristics of asthma control, severity [37] and response to oral bronchodilators [38].

### Radiographic identification of small airways inhomogeneity

High-resolution computed tomography (HRCT) methodology has permitted a non-invasive direct radiographic assessment of the luminal caliber and wall thickness of medium and large airways of >2mm in diameter that is reproducible and related to clinical disease severity [39, 40]. However, while the limits of resolution of HRCT imaging do not currently allow direct assessment of the small airways, one can indirectly evaluate changes in airways of < 2mm in diameter through measures of changes in regional air trapping, indicated by areas of low lung attenuation of ≤900 Hounsfield units. With the asthma subject performing a suspended breath hold at RV, areas of low attenuation can be visualized intermixed with areas of higher attenuation, creating a black and white mosaic pattern. Low lung attenuation regions, in black, represent air trapped behind functionally closed peripheral airways and are easily differentiated from high attenuation lung regions, which appear white, from which air has been normally expelled through patent airways. Changes in air trapping can also be assessed within regions of the lung under different physiologic conditions such as after bronchodilator or after allergen or methacholine challenge [41]. Radiation exposure remains a limitation of HRCT.

An advantage of magnetic resonance imaging, another non-invasive procedure, is the absence of ionizing radiation exposure. However, the conventional hydrogen based procedure is of limited value for viewing distal airways because of poor contrast resolution. This problem can be largely solved by the use of inhaled [3] He, which allows direct visualization of the lung airspaces, analysis of ventilation distribution, estimated non-ventilated lung volume and percentage of diseased lung. Inhaled [3] He distributes evenly in the healthy lung, with the airspaces appearing a homogeneous white in color. In the presence of obstructive airways disease, early airway closure results in airspaces distal to the obstruction appearing black in color, as they are unable to fill with [3] He. These ventilation defects correlate with FEF_25-75_ values and thus may estimate small airways disease [42].

With the recognition that inflammation of the distal airways plays a significant pathophysiologic role in asthma, there have been increasing efforts to develop a non-invasive, reliable and reproducible means of assessing small airways disease and its response to therapy. To date, while many techniques appear promising, no single procedure has proven to be the “gold standard” that provides an unrefuted correlation with lung pathology and clinical outcomes. It is therefore probably most practical, at this time, to employ some combination of studies that could include spirometry, plethysmography, nitrogen wash out, FeNO, impulse oscillometry, HRCT and/or [3] He magnetic resonance imaging to best assess distal airways disease, until future research will make a more comprehensive, evidence based approach a reality.

## Small airways in severe asthma

About 5–10% of patients with asthma are deemed to have severe disease, defined by the European Respiratory Society and the American Thoracic Society as “asthma that requires treatment with high dose inhaled corticosteroids (ICS) plus a second controller and/or systemic corticosteroids to prevent it from becoming uncontrolled or that remains uncontrolled despite this therapy” [43]. Treatment noncompliance, failure to use inhalers correctly, heterogeneity of disease phenotypes and comorbidities are the main contributing factors to poor asthma control. To date, there is no universal agreement as to the classification of phenotypes and endotypes that define this multifactorial disease. A popular, relatively simplistic classification is dependent on the characteristics of airways inflammation. Type 2 inflammation, either driven by Th2 lymphocytes, as is typical of atopic asthma, or type 2 innate lymphoid cells as can be seen in adult onset non-atopic asthma, is characterized by eosinophilic inflammation [44]. In contrast, non-Type 2 asthma may be characterized by a predominance of neutrophils, a mixed granulocytic pattern, or a paucicellular pattern [45]. Eosinophilic asthma is evidenced in approximately 50% of asthma patients and is generally more responsive to ICS therapy than is non-Type 2 disease [46, 47]. It is hoped that the development of reliable biomarkers that reflect underlying pathobiological processes in asthma, potentially including blood eosinophils, FeNO, IgE, periostin, dipeptidyl peptidase 4 and allergen specific IgE testing would help to accurately identify patient phenotypes, in an effort to best tailor therapy to improve asthma outcomes. However, use of these biomarkers to drive therapeutic decision-making has been of yet incompletely successful. Considering the ineffectiveness of systemic inflammatory biomarkers to entirely predict therapeutic response to corticosteroids, it is possible that structural changes such as small airways disease in asthma may an important and relatively overlooked phenotype. For those patients with predominant small airways disease, it is possible that sub-optimal outcomes are the result of sub-optimal drug delivery to the small airways.

A definitive assessment of the impact of small airways disease on severe asthma is limited by the lack of a practical gold-standard determinant of the small airways. However, using the aforementioned surrogate markers of small airways disease, including physiologic assessments and imaging techniques, many published studies implicate small airways dysfunction in increased symptoms, risk of exacerbations, and asthma severity. Measures of small airways disease can change with asthma treatment and correlate with asthma control [48]. Further, small airways dysfunction is implicated in asthma severity of both adults and children.

Usmani and colleagues performed a systematic review to determine the prevalence of small airways disease in adult asthma. Fifteen studies were identified, which utilized a variety of techniques including spirometry, plethysmography, nitrogen washout, impulse oscillometry, and high-resolution computed tomography to assess the small airways. Overall estimates determined small airways disease affects 50-60% of individuals across the range of persistent asthma severity [49]. In children with asthma, a majority suffer from small airways disease, determined using FeNO and nitrogen washout [50].

Autopsies from fatal asthma showed intense inflammation, small airway structure abnormalities and luminal plugging in peripheral airways [2]. The question remains if the extent of these changes reflects the pathogenesis of the underlying severe disease versus it reflects the severity of acute phase only. Other studies looking at inflammation in distal airways of severe asthmatics found a more severe and possibly qualitatively different form of inflammation when compared to non-severe asthmatics. Balzar et al. examined transbronchial biopsies from difficult-to-treat severe asthmatics, finding a higher total number of inflammatory cells, yet no qualitative percentage changes among small airways when compared to large airways [51]. Wenzel et al. compared the inflammation pattern of small airways in severe and moderate asthmatics [52]. Neutrophils were more pronounced in bronchoalveolar lavage, and transbronchial and endobronchial biopsies from severe asthmatics compared with the less severe group. The extent to which airway neutrophilia is a primary process, or a secondary effect of glucocorticoids improving neutrophils’ survival and reducing apoptosis continues to be an area of interest. Distal airways are a major site for airway remodeling in severe asthmatics. Dolhnikoff et al. found significant small airways remodeling with an increase in type collagen, matrix metalloproteinase and fibronectin and a decrease in collagen III localized to the outer part of the lung in patients who died of asthma [53].

Spirometric determination of small airways dysfunction correlates with clinical asthma outcomes. The NHLBI Severe Asthma Research Program (SARP) enrolled three cohorts of asthmatics, enriched for severe disease, who are extensively characterized. Within the first two SARP cohorts, small airway disease was assessed by FEF_25-75_ and compared by quartile. Quartiles with more severe dysfunction had more frequent asthma symptoms and higher healthcare utilization, and were characterized by higher eNO, IgE levels, and bronchial hyperreactivity [21]. Small airways dysfunction, again measured by reduction in spirometric measurement of FEF_25-75_, was also positively correlated with exacerbation frequency in both adults and children with exacerbation-prone asthma [54]. Similarly, children from a Boston cohort with reduced FEF_25-75_ have substantially higher risk of exacerbations and systemic steroid use compared with those who have normal lung function [18]. The extent to which small airways define asthma characteristics however is disputed as the relationships are not consistently reproducible [55].

Measurements of small airways disease through exhaled nitric oxide and oscillometry have also been associated with asthma severity and clinical outcomes. Van Veen et al. found higher levels of FeNO in difficult to treat, steroid dependent severe asthma compared to mild-moderate disease, and these measurements correlated closely to other measures of peripheral airway disease [29]. Further, FeNO correlated with rapid lung function decline over time, particularly in those with relatively conserved baseline lung function [56]. Berry et al. showed reproducibly higher alveolar NO concentrations in refractory asthma group compared to mild-moderate asthma group [57], supporting the hypothesis that refractory asthma is associated with distal lung inflammation. The high level of alveolar NO also correlated positively with BAL eosinophils, indicating an eosinophilic inflammation type in the lung periphery in refractory asthma. Keen et al. described a correlation between small airways FeNO and airways hyperreactivity in a pediatric subset of asthmatics [50]. Impulse oscillometry has also been used to assess uncontrolled asthma in children [58].

Kraft et al. examined the inflammation pattern in small and large airways in patients with nocturnal asthma and non-nocturnal asthma [59]; more CD4^+^ lymphocytes and eosinophils were noted in the alveolar tissue of nocturnal asthma group at 4 AM. This suggests that inflammation at peripheral small airways plays an important role in nocturnal asthma and worsening lung function at night.

## Drug delivery in severe asthma with small airways disease

Anti-inflammatory therapy with inhaled corticosteroids (ICS), with or without long acting B2-agonists (LABA), is the cornerstone of management of persistent asthma. Nonetheless, a significant number of patients do not gain optimal asthma control in spite of being treated with a high dose ICS/LABA combination [60], calling into question the ability of commonly used devices to deliver medication to the small airways. For individuals with small airways disease, and certainly for those with severe asthma, anti-inflammatory therapy needs to reach and address inflammation of the entire affected airway, and therefore efficacy of delivery becomes very important when selecting treatment [61].

Fundamentally, the efficacy of any topical inhaled medication is dependent upon successful distribution of the drug to the site of disease. Targeting small airways inflammation in severe asthma is critically important as the combined surface area of small airways far exceeds the surface area that large central airways provide. Pulmonary distribution of medication is impacted by the size of the inhaled particle, measured in mass mean aerodynamic diameter (MMAD). Fine ICS particles are defined as MMAD ≥ 2 microns in diameter and <5 microns, and are the product of inhaled therapy utilizing either a dry powder inhaler (DPI) or a hydrofluoroalkane (HFA) propelled suspension delivered via a metered dose inhaler (MDI). Extra-fine particle sized HFA solutions, with MMAD of <*2* microns delivered via MDI, have been more recently developed and are licensed for use internationally, however with variable approval for treatment of individuals 5–12 years of age. Comparative examples of inhaler mechanisms, MMAD and pulmonary drug distribution are listed in Table 2. Patient factors, including inhaler technique, use of a spacer device, inspiratory volume and flow rate, also impact medication delivery to the lungs and may be affected by the inhaler device.

**Table 2:** Comparison of particle size and lung delivery among selected inhaled corticosteroid therapies [82]

Drug	Formulation	Particle size (microns)	Lung deposition
Fluticasone DPI	Dry powder	5.4 μm	15%
Fluticasone HFA	Suspension	2.4 μm	13–18%
BDP- Modulite®	Suspension	2.6 μm	36%
BDP UF-HFA	Solution	1.1 μm	>56%
Ciclesonide HFA	Solution	1–2 μm	52%
Flunisolide HFA	Solution	1.2 μm	68%

*BDP* beclomethasone dipropionate, *DPI* dry powder inhaler, *HFA* hydrofluoroalkane, *UF* ultrafine

**Fig. 1:**
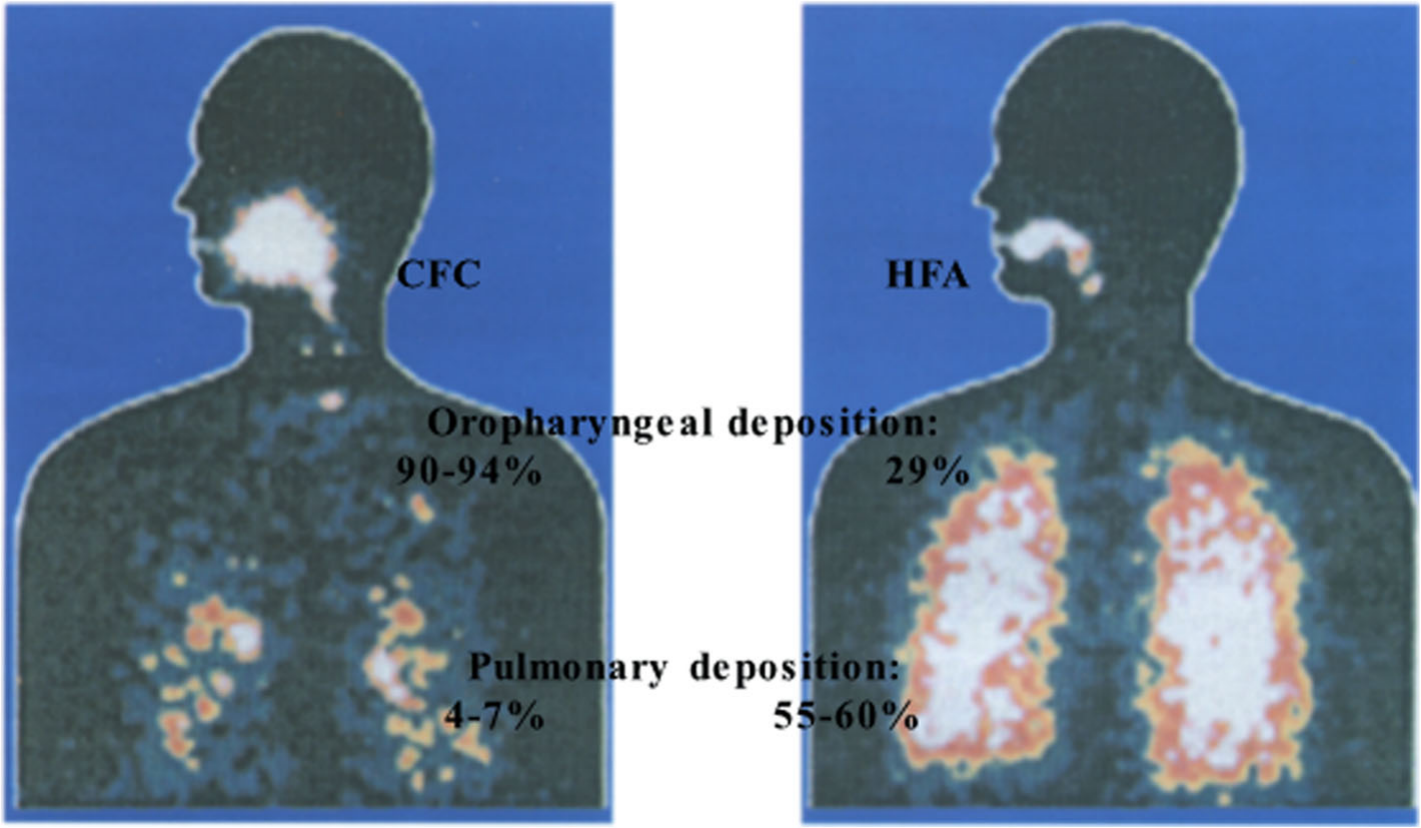
Lung Deposition of Beclomethasone diproprionate, Hydrofluoroalkane vs chlorofluorocarbon propellant inhaler [81]. Note the mosaic pattern of areas of low attenuation (representing air – which appears black – trapped behind diseased and functionally closed small airways) juxtaposed with areas of higher attenuation (whiter-appearing, representing lung regions from which the air has been normally expelled through patent airways)

Several studies have utilized imaging techniques to characterize and quantify the deposition of aerosols in the lungs. A landmark study using gamma scintigraphy compared HFA with CFC solutions [62]. In this study, an HFA inhalation showed ~40% oropharyngeal deposition, achieving 60% of the dose to the pulmonary tree including the small airways (Fig. 1). In comparison, the device using chloroflurocarbons had <10% pulmonary distribution, mainly in the large airways. Therefore, the dose of inhaled corticosteroid required to achieve improvement in lung function or other clinical outcomes will be higher in formulations with larger particle size. With regard to an effect on small airways, a CFC dose would need to be 3.2 times the HFA dose to result in an equivalent improvement in FEF_25-75_ [63]_._

Multiple practical surveys comparing extra-fine particles to alternative delivery methods have shown benefit in asthma control, quality of life, and lung function [64–69]. In moderate to severe asthma, combination ICS/LABA delivered in extra-fine particles had more impact on symptoms and exacerbations than the components delivered by CFC or DPI [70]. Extra-fine particle ICS also seem to improve airway hyperresponsiveness moreso than fine particle ICS [71]. Smaller particle size also improves pulmonary distribution of short-acting bronchodilator inhalation, however this may not lead to improved clinical efficacy [72].

Assessment of markers of inflammation of the small airways has supported the utility of extra-fine particle ICS inhalers in small airways disease. In one study addressing airways inflammation, of 12 patients with mild to moderate asthma, transbronchial and endobronchial biopsy specimens taken before and after 6 weeks of treatment with extra-fine HFA-flunisolide revealed significant reductions in interleukin 5, eotaxin, and eosinophils in both central and peripheral airways [73]. Further analysis from the same biopsy study looking at remodeling revealed a significant reduction in small airway expression of smooth muscle α-actin, which correlated with improved FEF25%-75%, but no effect on collagen deposition or expression of transforming growth factor β [74].

The noninvasive techniques utilized for assessment of small airways disease can also predict or measure response to therapy. For example, ventilation heterogeneity, measured by nitrogen washout, can predict clinical response to increasing ICS dose and loss of control from down titration [30]. Stable asthmatics with evidence of abnormal acinar airway function measured by nitrogen washout techniques may benefit from switching to an extra-fine ICS preparation [75]. Extra-fine ICS can reduce exhaled nitric oxide more than larger particle preparations [76]. Impulse oscillometry can identify decreases in small airways resistance with use of extra-fine ICS [77], even in the absence of significant spirometric change [78]. Finally, while not true for non-extra-fine ICS [79], HRCT imaging studies can show a relative efficacy of extra-fine HFA over CFC devices for reducing air trapping after methacholine challenge [41].

It is important to note that extra-fine particle ICS may be added to maximal doses of large particle ICS/LABA in severe uncontrolled asthmatics in an attempt to achieve control, without conferring significant additional risk of systemic adverse event [80].

The advent of extra-fine solution based ICS and ICS-LABA formulations appear to be an important advance in asthma therapy, particularly in view of evidence of small airways dysfunction in a significant proportion of asthma patients, despite non-extra-fine ICS treatment across all treatment steps of current guidelines. Studies demonstrating differences in asthma control, and measures of lung function, airway hyperresponsiveness, impulse oscillometry, nitric oxide diffusion, HRCT imaging and inflammatory markers, including alveolar and bronchial nitric oxide suggest that extra-fine-particle pressurized MDIs, by addressing airway function and inflammation in the distal as well as the proximal lung, appear to have additional clinical benefits in the treatment of asthma compared with non-extra-fine particle therapy. Additional studies are necessary to confirm and extend these findings and to assess the long-term benefit of these extra-fine inhaled MDI formulations on the clinical course of asthma, especially in patients with severe asthma who exhibit the small airway asthma phenotype. Further, data reporting safety, outcomes, and biomarker responses are lacking in the pediatric population.

## Conclusions

Small airways disease affects a majority of asthmatics across the spectrum of severity. The impact of small airways disease on asthma outcomes relates in part to its effect on lung function. While a gold standard for diagnosis does not exist, assessment of small airways disease can be made through a variety of noninvasive techniques. Importantly, however, individuals with small airways disease may benefit significantly from inhalers delivering extra-fine particles to those distal aspects of the lung. Evaluation of small airways disease in asthma, particularly for patients with severe asthma, may improve disease treatment, but the full benefit on long term outcomes is not yet clear.

## Unmet needs in severe asthma and small airways disease

Unmet needs:

1. A reliable index of noninvasive, widely available testing would allow easier diagnosis and monitoring of small airways disease in the primary care and specialty setting.
2. Additional studies need to assess the long term benefit of extra-fine particle inhaler therapy on remodeling, lung function, and other outcomes for individuals with small airways disease.
3. Despite an ever-increasing utilization of biologic medications for individuals with moderate to severe asthma, the benefit of these therapies for small airways disease has not been well established.
4. The impact of small airways disease in children with severe asthma is not well described. Further studies assessing the prevalence, clinical features, assessment, and effect of treatments on small airways disease in asthma should focus on the pediatric population.

## Abbreviations

CFC: Chloroflurorcarbon
FEF_25-75_: Forced mid expiratory flow between 25% and 75%
FeNO: Fractional exhaled nitric oxide
FEV1: Forced expiratory volume in 1 second
FVC: Forced vital capacity
HFA: Hydrofluoroalkane
HRCT: High resolution computed tomography
ICS: Inhaled corticosteroid
IOS: Impulse oscillometry
LABA: Long acting beta-2 agonist
MDI: Metered-dose inhaler
MMAD: Mass mean aerodynamic diameter
NO: Nitric oxide
NOS: Nitric oxide synthases
RV: Residual volume
SVC: Slow inspiratory vital capacity
TLC: Total lung capacity
